# The Role of KI67 in Predicting Post-ESS (Endoscopic Sinus Surgery) Outcomes in CRSwNP (Chronic Rhinosinusitis With Nasal Polyps)

**DOI:** 10.7759/cureus.79748

**Published:** 2025-02-27

**Authors:** Mihai I Tănase, Marcel Cosgarea, Raluca Maria Hendea, Peter L Ujvary, Maximilian Dindelegan, Gheorghe Doinel Radeanu, Alma A Maniu, Constantin Stan

**Affiliations:** 1 Department of ENT, “Iuliu Haţieganu” University of Medicine and Pharmacy, Cluj-Napoca, ROU; 2 Department of Otolaryngology, ”Iuliu Haţieganu” University of Medicine and Pharmacy, Cluj-Napoca, ROU; 3 Department of Anatomical Pathology, “Iuliu Haţieganu” University of Medicine and Pharmacy, Cluj-Napoca, ROU; 4 Department of ENT, "Iuliu Hatieganu" University of Medicine and Pharmacy, Cluj-Napoca, ROU; 5 Department of Surgery - Practical Abilities, “Iuliu Haţieganu” University of Medicine and Pharmacy, Cluj-Napoca, ROU; 6 Department of Surgical Clinical, “Lower Danube” University, Faculty of Medicine and Pharmacy, Galati, ROU; 7 Department of Otolaryngology, “Iuliu Haţieganu” University of Medicine and Pharmacy, Cluj-Napoca, ROU

**Keywords:** crswnp, endoscopic sinus surgery (ess), ki67, recurrence, snot 22 questionnaire

## Abstract

Introduction

Nasal and paranasal sinus inflammation, specifically chronic rhinosinusitis with nasal polyps (CRSwNP), is a prevalent condition often addressed through endoscopic sinus surgery (ESS). Despite the commonality of this surgical intervention, recurrence rates post-ESS remain significant. This study explores the relationship between the expression of KI67, a protein indicating cell proliferation, and the likelihood of recurrence in CRSwNP patients who have undergone ESS.

Methods

Thirty patients undergoing ESS for CRSwNP were enrolled in this prospective study conducted between January 2023 and December 2023. Nasal polyp tissue samples, obtained during the surgical procedure, were subjected to immunohistochemical analysis to determine KI67 expression levels. Post-surgical follow-up was conducted for a period of six months to monitor for recurrence, indicated by the reappearance of nasal polyps upon endoscopic examination.

Results

The average number of KI67-positive cells per high-powered field (HPF) was 63.7 (range, 21-82). Nasal polyp recurrence was observed in 9 patients (30%) within 6 months following ESS. A statistically significant difference in mean KI67 expression was found between patients experiencing recurrence and those who did not (74.3 ± 11.1 vs. 53.1 ± 11.6, p=0.003). Furthermore, a positive correlation emerged between KI67 expression and Sino-Nasal Outcome Test-22 (SNOT-22) scores (Pearson correlation coefficient, r=0.42, p=0.02).

Conclusion

The results of this study indicate that KI67 expression could potentially serve as a predictor of recurrence and disease severity in CRSwNP patients following ESS. Confirmation of these findings and determination of the clinical utility of KI67 as a prognostic marker will necessitate further investigation with an expanded sample size and extended follow-up period.

## Introduction

Chronic rhinosinusitis with nasal polyps (CRSwNP) is an inflammatory condition affecting the nasal and paranasal sinuses and is distinguished by the development of nasal polyps. These polyps can induce a variety of debilitating symptoms such as nasal obstruction, olfactory dysfunction, facial pressure, and recurring infections [1]. The pathogenesis of CRSwNP is intricate and multifactorial, encompassing a complex interaction of genetic predisposition, environmental influences, and immune system dysregulation. Endoscopic sinus surgery (ESS) represents a fundamental treatment approach for CRSwNP, with the objective of symptom relief and enhancement of quality of life [2]. Nevertheless, recurrence rates post-ESS remain considerable, with polyps frequently re-emerging months or years following the procedure. This highlights the necessity for dependable markers to forecast recurrence risk and inform individualized treatment approaches [3].

KI67, a nuclear protein, functions as a recognized marker of cellular proliferation. Its expression has been thoroughly investigated in the realm of oncology, where it is frequently linked to tumor aggressiveness and prognosis. Emerging evidence indicates that KI67 may also be implicated in inflammatory conditions, including CRSwNP. Research has shown elevated KI67 expression in nasal polyps in comparison to healthy nasal mucosa, implying a connection between cellular proliferation and polyp development. Moreover, some investigations have suggested a possible correlation between increased KI67 levels and a heightened risk of recurrence following ESS, underscoring its potential as a prognostic indicator [4].

The present study aims to examine the correlation between KI67 expression in nasal polyps and the probability of recurrence after ESS for CRSwNP. We hypothesized that elevated KI67 expression would be linked to a higher risk of polyp recurrence within a six-month postoperative period [5]. Furthermore, we aimed to investigate the correlation between KI67 expression and SNOT-22 scores, an established patient-reported outcome measure evaluating the severity of sinonasal symptoms [6]. By clarifying the role of KI67 in CRSwNP recurrence, this research could contribute to improved patient selection for surgery, tailored treatment strategies, and ultimately, more favorable long-term outcomes [7].

## Materials and methods

Ethics approval

This research was performed in compliance with the Declaration of Helsinki and was approved by the Ethics Committee of “U.M.F Iuliu Haţieganu” Cluj Napoca (approval number: 255/30.06.2021). Prior to participation, all patients provided written informed consent, and their data were anonymized to ensure confidentiality.

Study population

This prospective study enrolled 30 patients diagnosed with CRSwNP who underwent ESS at Cardiomed Hospital, Cluj-Napoca, between January 2023 and December 2023. The diagnosis of CRSwNP was based on a thorough assessment, incorporating a detailed medical history, endoscopic examination, and computed tomography (CT) scans of the paranasal sinuses. The detailed medical history encompassed an evaluation of symptoms, including nasal obstruction, nasal discharge, facial pain or pressure, and diminished olfactory function, along with the presence of comorbidities such as asthma and aspirin sensitivity. We also specifically noted the patients' history regarding smoking, asthma, and non-steroidal anti-inflammatory drug (NSAID) intolerance. Nasal endoscopy was utilized to examine the nasal cavity and identify nasal polyps, mucosal inflammation, and purulent discharge. CT scans served to determine the extent of the disease, including the degree of sinus opacification, mucosal thickening, and the presence of anatomical variations. Patients with cystic fibrosis, primary ciliary dyskinesia, or other systemic diseases known to impact the sinonasal tract were excluded. Patients were followed up for six months after surgery.

Surgical procedure

All patients underwent ESS performed by the same experienced otorhinolaryngologist. The surgical procedure adhered to standardized techniques and was designed to achieve adequate disease control, including nasal polyp removal, ethmoidectomy, and maxillary antrostomy where necessary. Polyps were excised using endoscopic instruments, with careful attention to minimizing trauma to the surrounding mucosa. The ethmoid sinuses were opened and cleared of diseased tissue, ensuring thorough removal of polyps and inflammatory debris. When indicated, the maxillary sinuses were accessed and cleared of pathological findings.

Tissue sampling

During the ESS procedure, nasal polyp tissue fragments were routinely collected as part of the standard surgical management of CRSwNP. No additional tissue was removed solely for the purposes of this study. The collected tissue samples were subsequently submitted for histopathological analysis, including an evaluation of KI67 expression.

Immunohistochemistry

Formalin-fixed, paraffin-embedded tissue sections were prepared from the collected nasal polyp specimens. Immunohistochemical staining for KI67 was conducted using the KI 67 (SP6) Rabbit Anti-Human Ki-67 Monoclonal Antibody (Clone SP6) kit (Ref.: MAD-000310QD/V, Vitro S.A, Spain) following the manufacturer's protocol.

Evaluation of KI67 expression

**Figure 1:**
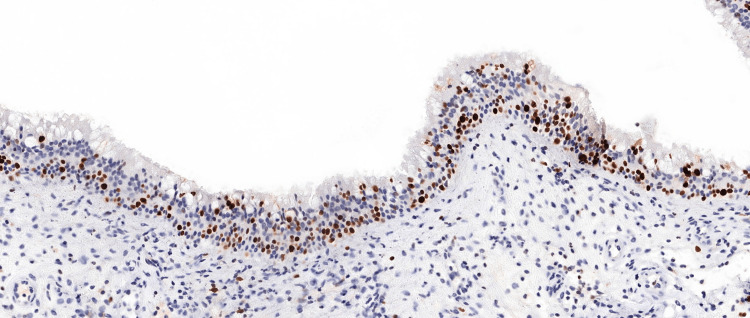
Immunohistochemical staining for KI67 in a nasal polyp specimen from a patient with recurrence after ESS Brown staining indicates KI67-positive cells (20x magnification). ESS: endoscopic sinus surgery

A single pathologist, blinded to clinical data, examined the stained tissue sections under a light microscope. The number of KI67-positive cells was counted in five randomly selected high-power fields (HPF, 400x magnification) per slide. Subsequently, the average number of KI67-positive cells per HPF was calculated for each patient (Figure 1).

Follow-up and assessment of recurrence

Patients were scheduled for follow-up appointments at six months post-ESS. In the six months after the surgery, the patients did not receive any medication except for nasal saline irrigation in the first two weeks. During each visit, a comprehensive endoscopic examination of the nasal cavity was conducted to evaluate for evidence of polyp recurrence. Recurrence was defined as the reappearance of nasal polyps, confirmed through endoscopic visualization.

Statistical analysis

Statistical analysis was conducted using SPSS software (version 25; IBM Corp., Armonk, NY, US). Descriptive statistics were employed to summarize patient characteristics and KI67 expression levels. The relationship between KI67 expression and recurrence was evaluated using the Mann-Whitney U test. The correlation between KI67 expression and SNOT-22 scores was examined using Spearman's rank correlation coefficient. A p-value of less than 0.05 was deemed statistically significant.

## Results

Patient characteristics

The study included a total of 30 patients with CRSwNP. The mean age of the participants was 42.5 years (range, 26-65 years) and 60% (n=18) were male. Table 1 summarizes the demographic and clinical characteristics of the study population. Notably, 50% (n=15) of the patients were smokers, 33.3% (n=10) reported alcohol consumption, 40% (n=12) had a history of asthma, and 33.3% (n=10) reported NSAID intolerance. The mean SNOT-22 score was 52.4, indicating a moderate impact of CRSwNP on patients' quality of life. The mean Lund-Mackay score, a radiographic measure of disease extent, was 18.5, suggesting moderate to severe sinonasal involvement.

**Table 1:** Patient characteristics NSAID: nonsteroidal anti-inflammatory drug; SNOT-22: Sino-Nasal Outcome Test-22; Lund-Mackay score: a radiographic scoring system for chronic rhinosinusitis

Characteristic	Value
Age	42.5 ± 12.3
Sex	
Male	18
Female	12
Smoker	
Yes	15
No	15
Alcohol Consumption	
Yes	10
No	20
Asthma	
Yes	12
No	18
NSAID Intolerance	
Yes	10
No	20
SNOT-22 Score	52.4 ± 10.8
Lund-Mackay Score	18.5 ± 3.2

KI67 expression and recurrence

**Figure 2:**
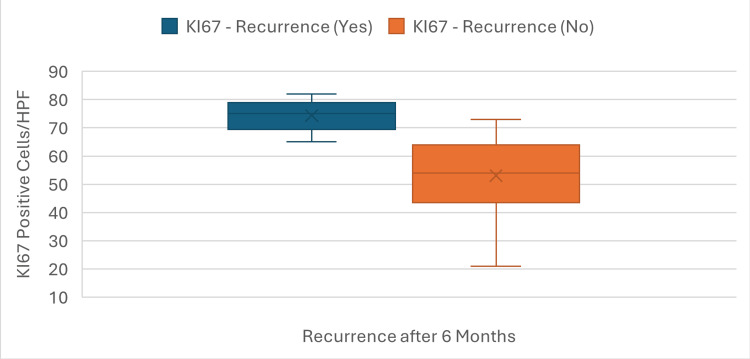
Distribution of KI67 expression in patients with and without recurrence

The mean KI67 expression in the nasal polyp tissue samples was 63.7 KI67-positive cells per HPF (range, 21-82). Nine of the 30 patients (30%) experienced recurrence of nasal polyps within 6 months following ESS. A significantly higher mean KI67 expression was observed in patients with recurrence compared to those without recurrence (74.3 ± 11.1 vs. 53.1 ± 11.6, p=0.003). This finding suggests that elevated KI67 expression in nasal polyps may be associated with a greater risk of recurrence after ESS (Figure 2).

**Figure 3:**
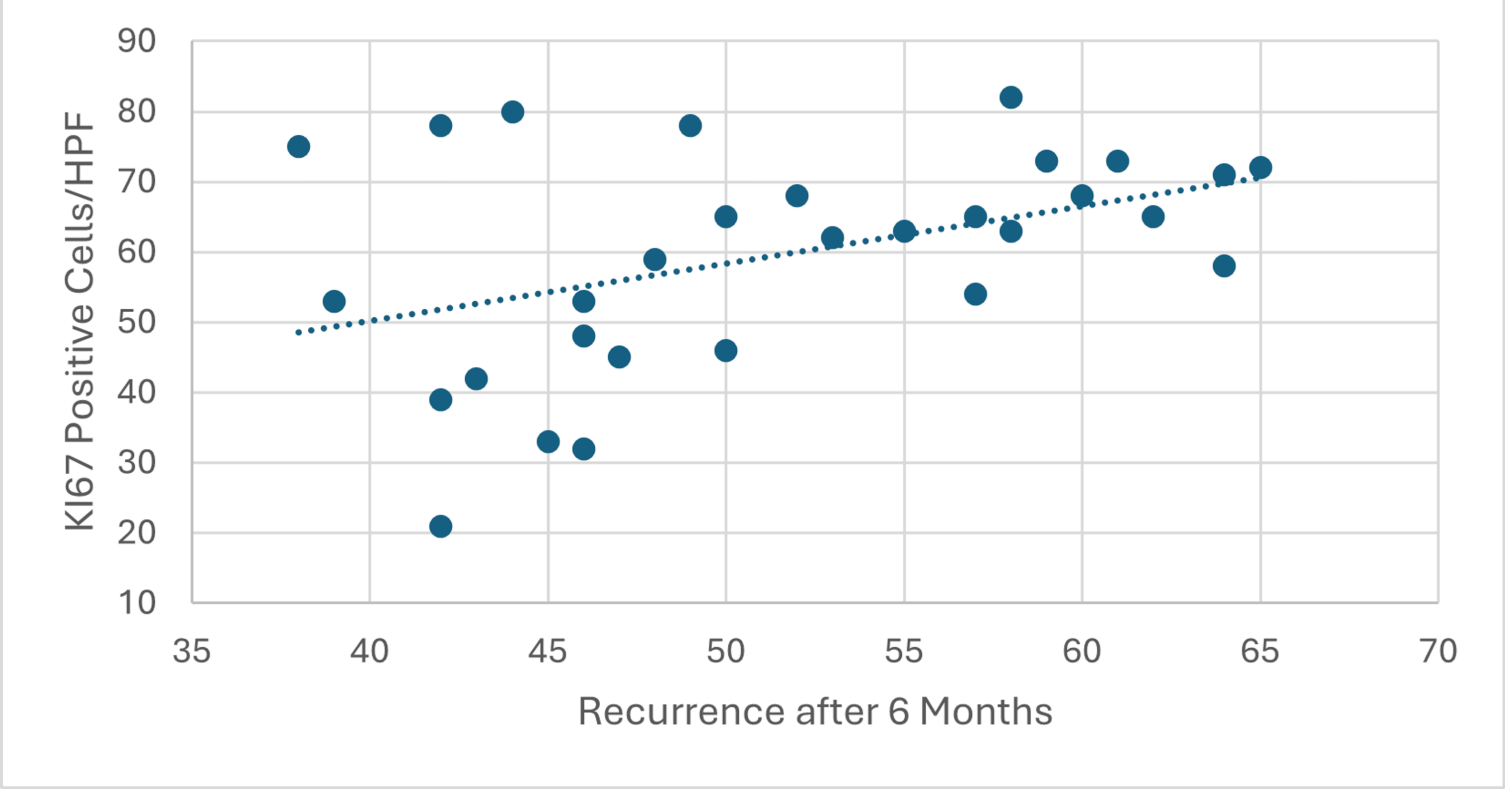
Correlation between KI67 expression and SNOT-22 scores

A positive correlation emerged between KI67 expression and SNOT-22 scores (Pearson correlation coefficient, r=0.42, p=0.02). This indicates that patients with higher KI67 expression tended to have more severe sinonasal symptoms, as reflected in their SNOT-22 scores. This correlation suggests that KI67 expression may be associated not only with recurrence risk but also with the overall severity of CRSwNP (Figure 3).

## Discussion

This study's findings suggest that KI67 expression in nasal polyps could potentially predict recurrence after ESS for CRSwNP. Patients experiencing recurrence within six months post-surgery showed significantly higher KI67 expression in their polyp tissue than those who remained recurrence-free [8]. This aligns with previous research investigating KI67's role as a prognostic marker in CRSwNP [9]. The biological rationale behind this association is rooted in KI67's function as a cell proliferation marker. Elevated KI67 expression signifies a larger proportion of actively dividing cells, potentially contributing to faster polyp regrowth post-surgery. Increased cell proliferation might also indicate a more aggressive inflammatory process or a greater tendency for tissue remodeling, both predisposing factors for recurrence [10].

Furthermore, a positive correlation between KI67 expression and SNOT-22 scores was observed, implying that increased KI67 levels might also correlate with greater disease severity. This aligns with the concept that heightened inflammation and tissue remodeling, indicated by elevated KI67 expression, could result in more pronounced symptoms and a more substantial impact on patients' quality of life [11].

Beyond its potential as a prognostic indicator, KI67's use as a biomarker is promising from a cost-effectiveness perspective. Immunohistochemical evaluation of KI67 is relatively inexpensive and widely accessible, already routinely used in many pathology labs. Compared to complex molecular tests or imaging studies, a KI67 assessment could offer an easily accessible and affordable method for risk stratification in CRSwNP patients [12]. This could be particularly advantageous in resource-limited settings, allowing clinicians to identify high-risk individuals who might benefit from closer monitoring or more intensive treatment strategies, ultimately leading to improved resource allocation and patient outcomes [13].

Although these results are encouraging, it is crucial to acknowledge the limitations of this study. First, the relatively small sample size may restrict the generalizability of the findings [14]. Second, the six-month follow-up period was limited; longer-term studies are necessary to assess KI67's predictive value for recurrence beyond this timeframe. Third, this study focused solely on KI67 expression and did not explore other potential biomarkers or clinical factors that might contribute to recurrence risk [15]. Furthermore, the single-center nature of the study could limit the generalizability of the findings to other populations. Finally, the study lacked a control group of patients without CRSwNP, which would have allowed for a more robust comparison of KI67 expression levels [16].

Despite these limitations, this study contributes valuable insights into the potential role of KI67 as a prognostic marker in CRSwNP. If validated in larger, long-term studies, KI67 could potentially be incorporated into clinical practice to identify patients at higher risk of recurrence after ESS. This could facilitate personalized treatment strategies such as closer follow-up, more aggressive medical management, or earlier consideration of revision surgery [17].

Future research should prioritize validating these findings in larger cohorts with longer follow-up periods. Moreover, investigating the interplay between KI67 expression and other potential biomarkers, as well as clinical factors like patient age, disease severity, and comorbid conditions, could further enhance our understanding of CRSwNP recurrence and improve patient care [18].

## Conclusions

This pilot study offers preliminary evidence supporting the potential role of KI67 as a prognostic biomarker in CRSwNP. Our findings suggest that KI67 expression in nasal polyps may be a valuable indicator of recurrence risk after ESS, potentially reflecting a more aggressive disease phenotype with a higher proliferative capacity. The observed association between KI67 expression and SNOT-22 scores further suggests that KI67 may also correlate with disease severity and symptom burden. These findings contribute to the growing body of evidence indicating that KI67 may play a crucial role in the pathogenesis and progression of CRSwNP, potentially guiding treatment decisions and personalizing patient care.

However, these results should be interpreted cautiously due to the study's limitations, including the small sample size, short follow-up period, and single-center design. Larger, well-designed studies with longer follow-up durations and diverse patient populations are needed to validate these results and establish the clinical utility of KI67 as a prognostic marker in CRSwNP. Future research should focus on validating these findings in diverse populations, exploring the underlying mechanisms linking KI67 to disease recurrence, and investigating the potential of KI67 as a therapeutic target. If confirmed, KI67 could be integrated into clinical practice to enhance long-term outcomes for patients.
